# Annotations

**Published:** 1893-06-03

**Authors:** 


### Annotations.

**Pains.** One of the greatest of all mysteries is pain. A thing so universal must, one would think, have a purpose in it. Pain may have several purposes. One at least is very evident: pain acts as a spur, and sometimes an extraordinarily sharp one. Ten thousand necessary things are done every day, both by men and animals, which would not be done if there were no pains to follow the neglect of them. We do not quarrel with pain the spur; our conflict is with pain the tormentor, pain the disabler. Hospitals are the civilised man's protest against disabling, unbearable pain; his defensive army which he employs to make war upon physical injury and disablement. Pain will never be eliminated from human experience so long as we are endowed with our present nervous system, but it may be reduced to a minimum. Disablements also may be reduced to a minimum. The amount and severity of the pains experienced and witnessed in hospitals is almost overwhelming. The accidents, counted by hundreds of thousands in London yearly; the fevers reckoned by tens of thousands; the chronic diseases, such as cancer, demanding its thousands of victims annually, and consumption demanding its thousands more; the destructive and agonising diseases which attack children by tens of thousands, with an infinite number of other unnamed ills, constitute an aggregate of distress and anguish which paralyses the imagination and makes realisation impossible. All these ills afflict the civilised man more sharply than the savage. They threaten the progress of civilisation itself. One of the problems of the immediate future is this: Can we stand up with physical force and resisting power sufficient to bear the strain which the intensifying struggle for survival will speedily put upon us? This question must be answered in the hospital: it must be answered by medical science. If medical science cannot answer it in the affirmative, it cannot be answered in the affirmative at all. Hospitals and medical science stand between civilised man and destructive pains and ills, like a strong army defending the fatherland. Is the army strong enough? If not, let us promptly make it stronger.

**Brains.** Civilised man always employs his best brains to do battle against his greatest obstacles. The modern man is very conscious of his physical ills. The most progressive, the most ambitious, the most imperious feel these difficulties most. The dull man cares nothing for obstacles; his heart fails at once, and he surrenders to the enemy. But the capable and the resolute hates lions in his path, and sets himself by boldness or stratagem to destroy them. What would the world not give for doctors who could cure all physical ills immediately, and guarantee an unfailing capacity for work and enjoyment throughout life a full century long? There is an extraordinary demand for capable doctors; never was there such a demand. And never was there such a supply. Nowhere is there to be found in Great Britain or in Europe a more striking aggregation of capable men and women than in the great hospitals! There the chemist vies with the physiologist; the physician with the surgeon; the nurse with both; and all with one another, in a strenuous tournament of devotion to the relieving of pain, the saving of life, and the restoring of limb and health. We computed some time ago that there were not fewer than seven hundred doctors associated with the various London hospitals; and the majority of these are picked men. There are probably not less than two thousand nurses; and they are the flower of their class for brains and character. All these are, of course, working for themselves and the world; but they are devoting themselves in the first instance with the most ardent and unremitting energy to the cause of the sick poor. Through the hospitals they pass to recognition; through the hospitals they ascend to competence and fame. The hospitals are the lower rungs of the ladder for them; and all men know how tremendously a man strives to acquit himself well when he is still at the beginning of his career. Whatever the human intellect can do is done at hospitals for the wounded and disabled in life's battle. Doctors and nurses supply an extraordinary abundance and competence of brains. Will not a generous and appreciative public supply a corresponding competence of material wealth?

**Gains.** If civilisation is a gain, skilled medicine is a gain. If science is a gain, the science of physiology is a gain. Measuring up our gains of the present century, is there any aggregate which can be considered of more practical worth than the aggregate gains of physiology and medicine? Do we need to mention more than the discovery of anæsthetics and the elaboration of antiseptic medicine and surgery? Those are large gains; of smaller ones we might count up hundreds. What ignorances and stupidities have modern doctors not put away which their fathers practised! How are every resource and detail of experience and highly-trained art placed at the service of the poorest and the meanest? Compare the modern hospital ward with the poison-house of a hundred years ago. Compare the modern hospital nurse with the hospital nurse of fifty years ago. Compare the medical student of to-day—the earnest, cultured, science-loving disciple of an earnest, broad and cultured science of medicine—with the pleasure-seeking, uncultivated, unambitious hospital-walker of the beginning of the century! A revolution has effected itself. Last century medicine was the last and least honoured of the scientific callings; to-day she is the most honoured and easily first. We do not yet know the gain which has accrued to modern man by fifty years' scientific study and practice of medicine. The doctor has revealed to mankind the new world of the microscope; he has expanded human knowledge indefinitely. Looked at with the eye of the physiologist man is more mysterious, more terrible, greater, and more noble than philosopher or priest ever saw him to be before. The modern world is the world of the physiologist and the scientific physician. All men are now using the doctor's eyesight. Every problem of man demands the physiologist's solution before it can be solved at all with any degree of completeness or permanence. All these gains have had their origins in hospitals. These and still greater gains will claim hospitals as their source and starting point. The seed of all future developments in medicine is sown in hospitals. If the world desires to be strong, to be healthy, to be filled with energy for the great struggles of the present and the future, it must with open-handed and ever-increasing generosity maintain the efficiency of its hospitals.

